# Bystanders and the Murder of George Floyd: Analyzing Bystander Intervention in the Course of a Police Killing

**DOI:** 10.1037/amp0001531

**Published:** 2025-05-12

**Authors:** Mark Levine, Chris Walton, Richard Philpot, Tina Keil

**Affiliations:** 1 Department of Psychology, Lancaster University

**Keywords:** George Floyd, bystander, intervention, policing

## Abstract

Using a detailed transcription, obtained from body-camera, CCTV, and smartphone footage of the murder of George Floyd, we examine the behavior of bystanders as events unfolded. Analysis reveals 205 direct verbal bystander interventions comprised of five forms (declaratives, assessments, interrogatives, imperatives, insults). We also describe the key physical intervention strategies deployed by the bystanders. We show that bystanders prioritize interventions based on what they “know” (rather than asking questions or making demands). We suggest that this is because assessment-based strategies are less likely to be seen as a direct challenge to the power of the police and therefore have more chance of inducing constructive engagement. Although bystanders were ultimately unsuccessful in persuading the police to change course, we identify five moments in the action sequences where the assessment concerns of the bystanders were taken up by the officers—albeit fleetingly. We argue that these bystander interventions create the opportunity for officers to break the pattern of behavior that will lead to murder. It is a failure of the officers and not the bystanders that the police are unable to take those opportunities. We argue that assessment-based interventions have the potential to breach structural and situational power dynamics that usually lead to bystander interventions being overridden or ignored. We conclude by drawing some wider implications for the way bystanders and police officers can be trained to improve the safety of individuals caught up in police arrests.

## Public Significance Statement

The United States faces an epidemic of police killings. These killings often take place in the presence of third-party bystanders. How do bystanders act when faced with police violence? We consolidated 12 separate in situ video/audio recordings of the murder of George Floyd—captured by body-worn cameras, bystanders’ smartphones, and CCTV—to create a detailed, moment-by-moment transcript. Analyzing this multimodal, multiperspective data, we identify the range of verbal and physical strategies that bystanders deployed to try and save George Floyd’s life. Our microbehavioral analysis reveals the complexity and coordination of third-party interventions in the trajectory of police violence. We demonstrate that bystander verbal strategies based around assessments (rather than question asking or making demands) have greater potential for getting the police to acknowledge the concerns of the bystanders. We show how bystander interventions were taken up by the police—albeit fleetingly—and that it was a failure of the officers and not the bystanders that meant that the lethal trajectory was not averted.

## Critical Moments: Bystanders and the Murder of George Floyd

George Floyd was murdered by Minneapolis police officer Derek Chauvin early in the evening of May 25, 2020. The widespread revulsion at videos of a White police officer kneeling on the neck of a Black man (for 9 min and 29 s) as he lay face down in the street, with his hands cuffed behind his back, complaining repeatedly that “I can’t breathe,” gave new impetus to the “Black Lives Matter” campaign. The murder ignited protests against systemic racism and police brutality in America and around the world.

Recent scholarship has begun to document some of the social, cultural, and political implications of the murder and its aftermath. For example, there have been studies of the emotional and mental health impacts of the case on people in the United States (Eichstaedt et al., 2021; Kim et al., 2024), on the differential effects the killing had on White and Black communities in the United States (Onwuachi-Willig, 2024; Sinclair & Starck, 2021; Sullivan et al., 2021), on the mental health of young people (Howard et al., 2023), on relations between the public and the police (Brantingham et al., 2022), on the nature of policing (Koslicki, 2022), on the effectiveness of protest (Reny & Newman, 2021), on the role of social media (Nguyen et al., 2021), and on the nature of institutions (e.g., Dreyer et al., 2020; Knopf et al., 2021).

### Bystanders and the Murder of George Floyd

One aspect that has yet to receive much scholarly attention is what the events surrounding the murder can tell us about the nature of bystander behavior. This is somewhat surprising given the pivotal role played by bystanders in creating and uploading the smartphone footage that went viral in the first place. Moreover, the footage reveals repeated attempts by bystanders (who were all strangers to each other and to George Floyd) to intervene with all four police officers present (not just Chauvin) as the terrible events unfolded. In fact, scrutiny of the actions of the bystanders was a key feature of the Chauvin murder trial (Gersen, 2021). For the prosecution, the bystanders were presented as witnesses to a murder they were trying to prevent. For the defense, bystander actions were challenged (from the perspective of the police) as potential threats to civil order and the safety of the officers. How then should we understand the behavior of bystanders on that day? What can the current state of psychological knowledge about bystander behavior contribute to understanding how the events unfolded? What more can be learned from a detailed, moment-by-moment examination of the actions of the bystanders on that day?

One of the distinctive features of the murder of George Floyd was how much of the episode was captured on video and how many different viewpoints of events became available. The first video to reach public consciousness was taken by the 17-year-old bystander Darnella Frazier, who had arrived outside the Cup Foods store with her 9-year-old cousin, just as Floyd was being restrained on the ground by the officers. Her video lasts 10 min and 9 s and captures Floyd saying: “I can’t breathe” (20 s into the clip) and then most of the period that Chauvin knelt on Floyd’s neck. It was this video, uploaded to Facebook and Instagram, that went viral and sparked the first of the protests. Later, footage from other bystanders, from CCTV cameras covering the street, and from the body-worn cameras of the police officers involved in the episode was also released to the public. This rich source of evidence, with video from multiple perspectives, from very close to the action with audio of what was said, as well as a “birds-eye” perspective on the action from CCTV cameras, created what Nassauer and Legewie (2021) referred to as the “optimal capture” required for high-quality video data analysis. This multimodal, multiperspective, triangulated data source provides an unprecedented opportunity to identify not only what happened when (Philpot et al., 2019) but also how the words and actions of each of the participants relate to each other.

### Ethics and Responsible Research

Of course, this means that we are also confronted with the trauma of George Floyd’s struggle as he pleads with the officers to “let him breathe” and then witness his death as he falls silent. There are clear ethical, psychological, and political challenges in approaching this kind of material. Researchers have a responsibility to respect the dignity of any person who dies at the hands of the police and to ensure that if the episode is to be analyzed, then any research work is consistent with the interests of the person who died. There are also responsibilities to the wider community to ensure that the research itself is not exploitative or at risk of traumatizing or undermining Black communities who recognize their own experiences in the suffering experienced by “a dying Black body” (Ibrahim, 2022).

This article seeks to meet those responsibilities by focusing on the actions of bystanders in the kinds of emergency events that can lead to death at the hands of the police. Recent analysis suggests that bystanders are present at around 44.5% of deaths at the hands of the police (Levine et al., 2024). Thus, the aim of this analysis was to explore how bystander dynamics play out in these kinds of situations, how they are related to what the police do (and do not do), and how these interactional sequences might help us understand the outcome. We know from the outset that there were multiple attempts by the bystanders to intervene in George Floyd’s murder but that these interventions were ultimately unsuccessful. The bystanders were unable to get the police officers (and Chauvin in particular) to stop what they were doing and to check on the well-being of George Floyd while he was under restraint. However, this outcome was not predetermined. Rather, it was the endpoint of a process that might have taken a different turn at different points in the sequence. Our aim in conducting the analysis was to see if there are key insights about individual bystander behavior, about bystander interactions with each other, about bystander interactions with the police, and about police interactions with each other that can be derived from this case and which might have led to a different outcome. Moreover, we seek to use the detailed microanalysis of the George Floyd’s case to examine the utility of our current psychological understanding of bystander behavior more generally. It is widely recognized that bystanders are a key resource in violence prevention (Levine et al., 2020; Nagin et al., 2023) and that more detailed understanding of real-life incidents is key to improving the utility of current bystander-focused violence prevention initiatives.

### What We Know About Bystander Behavior in Emergencies

There is surprisingly little research on how bystanders intervene in real-life emergencies (Philpot et al., 2020) and whether and how they coordinate with others when they do (but see Au-Yeung et al., 2024; Ejbye-Ernst et al., 2022, for exceptions). Instead, most of the work explores whether an individual bystander is likely to intervene in the first place and the negative impact that the presence of others is likely to have on that decision. This traditional approach is often referred to as the study of “the bystander effect” (Latané & Darley, 1970). Here the idea is that people are more likely to intervene when they are on their own than when they are with others. However, a decade-old meta-analysis (Fischer et al., 2011) demonstrates that the bystander effect does not hold for violent or dangerous emergencies (like the George Floyd case) and indeed that the presence of others can result in a “reverse bystander effect,” namely, an increased likelihood of intervention in the presence of others than when alone. More recent work analyzing CCTV footage of public aggression and violence in three countries (Netherlands, South Africa, and the United Kingdom; Philpot et al., 2020) shows that some bystander intervention is highly likely to occur (in 9 out of 10 incidents).

This still does not tell us about *how* the bystanders are likely to intervene and how that intervention might be coordinated with the others at the scene of the emergency. It also does not tell us what makes some interventions successful while others fail. Early research using CCTV footage of 42 incidents in the United Kingdom suggests that successful bystander intervention is likely to be cumulative, with at least three bystanders contributing to the sequence of interventions (Levine et al., 2011). Bystander interventions are also more likely to be attempts to deescalate rather than escalate the emergency. These ideas are developed further in qualitative analysis of bystander interventions, which identify “caring collectives” (Bloch et al., 2018) and “circles of peace” (Weenink et al., 2022) as key to successful bystander intervention. Research from a social identity perspective on group processes in bystander behavior (Levine & Manning, 2013) argues that it is the psychological relationship between bystanders and all those present at an emergency that is key to how the action unfolds. This theoretical lens points to the importance of understanding both intra-group and intergroup dynamics in emergency settings. It seeks to explore the psychological relationships between bystander and victim, between bystander and fellow bystander, and between bystander and perpetrator in interpreting how bystanders might act.

To the best of our knowledge, there is no detailed work on the complexities of bystander intervention in the context of police aggression and violence. There are studies of police and community violence as part of intergroup conflicts (e.g., Gerber et al., 2018; Stott & Radburn, 2020; Stott & Reicher, 1998). These studies usually focus on events involving the participation of large numbers of people in intergroup encounters around sports matches, political protests, or other collective activities. However, in the murder of George Floyd, we have a more “classic” bystander case, in that the original incident was not part of an explicit intergroup conflict. The police were called because of a claim that Floyd had used a counterfeited bill to pay for cigarettes in the Cup Foods store. The bystanders who witnessed the police attempts to arrest Floyd did not know him or indeed each other. The situation is therefore much closer to the more anonymous public space emergency familiar to bystander researchers, with the added complication that the police, one of the parties, are both the source of the emergency and the holders of the monopoly on legitimate violence.

### Verbal and Nonverbal Bystander Interventions

For the purposes of the analysis that follows, we created a detailed transcription of the action sequences from the first bystander involvement to the time that George Floyd is put on the ambulance stretcher. The transcript was created out of the stitching together of different sources of audio and video footage—including the audio and video feeds from the body camera of officer Lane (which we used as the baseline)—synchronized with the feeds from the bodycams of officers Keung and Thao and augmented by mobile phone footage generated by the bystanders Darnella Frazier, Alyssa Funari, and Genevieve Hansen. This triangulation of audio and video recordings allowed the accuracy of the transcript to be checked against multiple sources and facilitated accurate transcription of utterances that would not have been audible from Lane’s audio alone.

The transcription employed the system developed by Jefferson (2004) for a conversation analytic approach to talk in action and included information not only about who says what, and in what order, but also the measurement and notation of pauses, overlaps, emphasis, speed, pitch—as well as relevant physical or emotional information conveyed in the face or the body.

To conduct the analysis, we defined bystander interventions as those verbal and nonverbal actions that orient to the well-being and/or treatment of George Floyd and which might function in some way to *directly* alter the behaviors of their intended recipient(s). Utterances wherein bystanders were accounting for a subsequent or prior intervention (e.g., by claiming status or expert knowledge, e.g., “I’m a firefighter from Minneapolis”) were not regarded as interventions orientated at the direct well-being or treatment of George Floyd and were thus not counted as bystander interventions under this definition.

We use this transcript to identify:

• all attempts at direct verbal bystander intervention—including who the bystanders are orientating to when they intervene,
• the different kinds of verbal bystander strategies that are deployed and how they relate to each other,
• the key features of any physical bystander intervention that the bystanders engage in, and
• the nature of the response by police officers to these verbal and physical bystander interventions.

We know at the outset that the bystander interventions failed to save the life of George Floyd. However, by exploring the action sequences in detail as they unfold, we seek to identify any critical moments wherein the trajectory might have changed and how bystander actions might have created the possibility of change.

## Method

### Ethics

The present study received ethical approval from Lancaster University’s Faculty of Science and Technology Ethics Committee (Ref: FST-2023-3657).

### Data

**Figure 1 fig1:**
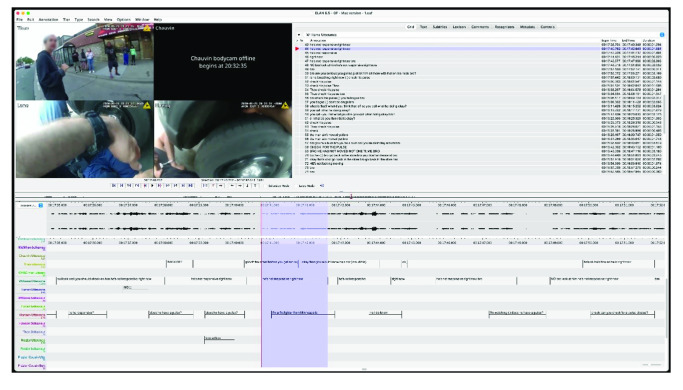
Time-Synchronized Split-Screen Master Video *Note*. GF = George Floyd. See the online article for the color version of this figure.

Data comprised video footage showing the interactions of the police, the victim, and members of the public during the restraint and murder of George Floyd on the evening of May 25, 2020, from 19:55 to 20:42 CST. In total, 12 publicly available videos were collated in March 2022 from social media platforms and websites. These videos were recorded on the body-worn cameras of the attending officers, the smartphones of bystanders, and local CCTV cameras. A full breakdown of these video sources and a temporal timeline of video capture are available on the Open Science Framework (https://osf.io/gdn9t/). These videos were amalgamated into a single master video, which showed four time-synchronized split screens covering multiple camera perspectives (see Figure 1). This offered comprehensive and detailed audio and visual data for transcription.[Fig fig1]

The initial transcript was based on the longest single continuous recording that was taken from the bodycam of officer Lane. Officer Lane’s bodycam captured video and audio from prior to his initial interaction with George Floyd through to discussions with his fellow officers after Floyd had been taken away in the ambulance. Lane’s audio and video, synchronized with the video from the bodycams of officers Keung and Thao, provided for the generation of a first-pass multimodal transcript in ELAN (2023; Version 6.5). The Elan transcript was then exported into MS Word to allow generation of a Jeffersonian (Jefferson, 2004) transcript, augmented by the examination of the synchronized audio recordings from the bodycams of officers Keung and Thao, and from the mobile phone footage generated by the bystanders Darnella Frazier, Alyssa Funari, and Genevieve Hansen. Despite these multiple audio and video streams, not all utterances were audible and transcribable, often owing to the number of people speaking at any one time, and not all speakers were identifiable.

### Participants

#### Bystanders

In total, eight bystanders were recorded as making utterances that were categorizable as interventions. The six bystanders who can be identified and whose utterances comprise the majority of the data set are (in the order in which they attempted to intervene) Charles McMillan, Darnella Frazier, Donald Williams, Alyssa Funari, Genevieve Hansen, and Kaylynn Gilbert. Other bystanders were present at various times, two made only one or two interventions, and others made no interventions.

#### Police Officers

Four officers were directly involved in the attempted arrest and killing of George Floyd. They were officers Lane and Kueng, who were first on scene and first attempted to arrest and restrain George Floyd, and officers Chauvin and Thao, who arrived on the scene at 20:16:45. Chauvin joined the restraint of George Floyd at 20:18:16 and began to kneel on Floyd’s neck at approximately 20:19:15. Officer Thao positioned himself at the rear of the police cruiser, between the sidewalk and his fellow officers and George Floyd.

## Analysis

### Verbal Bystander Interventions

In the 11 min and 49 s between Charles McMillan’s first words to George Floyd (the first verbal bystander intervention) and Floyd being placed on the stretcher, the bystanders made 205 utterances that are hearable as direct bystander interventions. Of these, 28 were orientated toward George Floyd and 177 toward the police officers.

The interventions were coded based on the initial part of the utterance, for example, “you enjoying it, look at you, your body language explains it you fucking bum” was coded as an assessment, though it clearly has an insult latched onto it. Interventions came in five different forms: declarative statements (utterances that provide information or which state facts), assessments (utterances that convey an evaluation of either George Floyd’s well-being or his treatment by the officers), interrogatives (utterances that are phrased as questions, both real and rhetorical), imperatives (utterances that tell the addressee to do something), and insults (utterances that disrespect or demean the addressee). There was also a *miscellaneous* category that contained utterances such as address terms, for example, “Bro,” and exclamations, for example, “Oh my god (bro).”

As shown in Table 1, the most frequent forms of verbal intervention were assessments (*n* = 65), followed by imperatives (*n* = 47) and interrogatives (*n* = 43). Declarative statements (*n* = 21) and insults (*n* = 9) are much rarer by comparison. The low count for insults, which might not accord with a casual hearing of the audio recordings, is a consequence of many insults being latched on to another form of utterance as in the above example.[Table tbl1]

**Figure 2 fig2:**
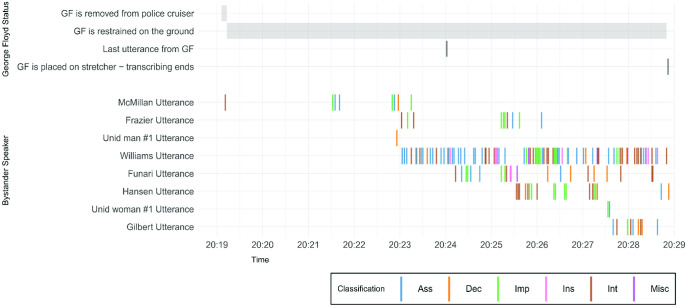
A Timeline of the Status of George Floyd and the Bystander Intervention Utterances Toward Police Officers (Classified by Type) *Note*. GF = George Floyd; Ass = assessments; Dec = declaratives; Imp = imperatives; Ins = insults; Int = interrogatives; Misc = single-word utterances (e.g., “bro,” “yeah”). See the online article for the color version of this figure.

Within and across speakers, and particularly for those speakers who made the greatest number of utterances, for example, Williams (106 utterances), McMillan (38), Frazier (29), Hansen (27), and Funari (22), there is a general pattern that sees speakers begin with declarative statements and assessments before moving to interrogatives and imperatives and only occasionally resorting to insults later in the sequence (See Figure 2).[Fig fig2]

What is also immediately obvious from the transcript is that, despite these numerous and varied attempts to engage with the police officers, there is very little by way of response or engagement with the attempted interventions. Officer Kueng responds only once, Chauvin responds twice, and Lane issues three responses; of those six responses, five relate to the management of the bystanders’ location, that is, instructions to the bystanders to remain on the sidewalk. By contrast, officer Thao, who is not directly involved in the restraint of George Floyd and whose primary responsibility may be glossed as “crowd management,” makes 49 responses to the bystanders. These can be broken down into minimal response tokens (e.g., “okay”), instructions to the bystanders to remain on the sidewalk (e.g., “get off the street before you get run over”), and attempts to counter the bystanders’ understanding of the situation, for example, by attributing Floyd’s situation to him being on drugs and thereby accountable for his predicament (e.g., “this is why you don’t do drugs kids”) or by challenging their assessments of Floyd’s well-being (e.g., “it’s hard to talk if you’r- if you’re not breathing”).

This failure of the police to act as a conversational partner reveals both the deontic order that shapes this interaction (Stevanovic & Peräkylä, 2012) and the key challenge faced by the bystanders. Both bystanders and police officers understand that members of the public do not have the right to intervene in the actions of police officers, and, indeed, to do so may be characterized as a criminal offence or bring violent sanction (Seigel, 2018). Both sides also recognize that officers have a monopoly on violence, and the police are under no obligation to account for their actions to others (Klockars, 1985). Engaging in a challenge to this deontic order is no easy matter.

The way bystanders seem to respond to this is by prioritizing the epistemic order as a counterweight. In other words, they try to ground their right to intervene in what they “know.” This footing goes some way to explaining why declarative statements and assessments are the more usual opening form of interventions by the bystanders. Rather than begin by questioning the officers, or making demands of them, they attempt to build a consensus around the illegitimate nature of the action itself. If they can establish—as public knowledge—that the actions of the officers are harmful or illegitimate, they can establish an epistemic-based right to intervene. Assessments (which are the most frequent form of intervention in the data) are key to this. A closer examination of the assessments reveals that they have two primary objects: George Floyd’s physical well-being and the legitimacy of the officers’ conduct. The assessments of George Floyd’s well-being reflect his worsening physical state over time, from “his nose is bleeding” to “he cannot breathe” and “he’s not responsive” to “he’s dead.” Assessments of the legitimacy of police conduct break down into those regarding the form of restraint being applied (“y’all know that is y’don’t gotta sit there with your knee on his neck bro”) and the necessity for it (e.g., “he ain’t even resisting arrest”).

Assessments also ground the other two major types of verbal interventions used by the bystanders: interrogatives and imperatives. Having opened with an assessment, typically, the bystanders use interrogatives before they use imperatives, that is, they assume a right to ask the officers questions about George Floyd’s well-being and their treatment of him before they assume a right to tell the officers to change their behavior. Following the pattern for assessments, the interrogatives typically have one of two subjects: the well-being of George Floyd (e.g., “does he have a pulse?”) or the legitimacy of the officers’ actions (e.g., “why’re you kneeing him even more?”). Given the general lack of a response from the police to their interrogatives, the bystanders either restate or upgrade their assessments or move to issuing imperatives (e.g., “GET THE FUCK OFF OF HIM”). The bystanders’ upgraded assessments are also driven by their shared understanding of George Floyd’s worsening physical state.

**Figure 3 fig3:**
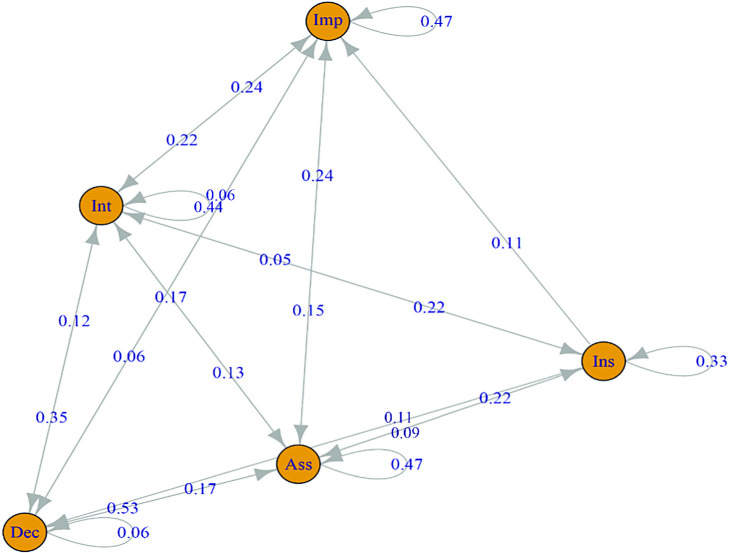
A Markov Chain Model of the Transition Probabilities in Utterance Prediction *Note*. Utterances directed by bystanders toward police officers. Imp = imperatives; Int = interrogatives; Dec = declaratives; Ass = assessments; Ins = insults. See the online article for the color version of this figure.

In seeking to explore the relationship between these different types of interventions more systematically, we ran Markov chain analyses on the sequential patterns of emergence (see Figure 3). Analyses of those utterances designed to alter the behavior *of the police officers* reveal that, with the exception of declarative statements, each utterance type is most likely to be followed by another utterance of the same type, for example, an assessment is most likely to be followed by an assessment (46.81%), an interrogative by an interrogative (43.9%), an imperative by an imperative (47.06%), and an insult by an insult (33.3%). This is, in part, a likely consequence of the failure of the police officers to act as conversational partners. It leads to repeated attempts from the bystanders to establish a connection using the current intervention form.[Fig fig3]

When it comes to transitions—or switches in bystander strategy from one form of intervention to another—again the role of assessments is central. Bystanders show a fairly balanced facility for switching from assessments to all other forms of intervention, declaratives (17.02%), imperatives (14.89%), interrogatives (12.77%), and insults (8.51%).

Interrogatives are most likely to transition to imperatives (21.95%) or to go back to assessments (17.07%). Imperatives are equally likely to transition to assessments and interrogatives (23.3%, each). The close relationship between declarative statements and assessments is clear—with a strong likelihood that each declarative statement will be followed by an assessment (52.94%) or an interrogative (35.29%). Transitions away from insults were too small in number to discern any reliable pattern.

Taken together, the volume, range, and adaptability of the verbal interventions that were made during the restraint and murder of George Floyd speak to the resilience and perseverance of the bystanders during this traumatic time.

### Physical Bystander Interventions

It is also evident from the video footage that bystander interventions are not limited to the verbal modality. Individually and collectively, the bystanders demonstrate nonverbal behaviors that, while not being attempts to intervene physically, may be considered interventions that are intended to alter the officers’ behavior. The first such behavior is the visible recording of the event using mobile phones. Chauvin first placed his knee on George Floyd’s neck, at 20:19:15 (on officer Lane’s bodycam). At 20:20:49, Darnella Frazier began recording. At the time that she started her recording, Frazier was walking past and away from the police cruiser; she had previously passed the scene moving in the opposite direction. Four seconds into her recording, she paused and moved back toward the scene but stopped such that the waste bin is positioned between her and the officers and George Floyd. Alyssa Funari started recording at 20:21:06. Both Frazer and Funari began recording before they said anything to the officers; Funari directed her first verbal intervention to the officers more than 3 min after she began recording. They were later joined in recording the situation by Genevieve Hanson who began recording at approximately 20:26:06. However, Hanson only begins recording *after* making several verbal attempts to intervene with the officers first.

The second form of nonverbal intervention is that of physical proximity to the officers and George Floyd. At no point did any of the bystanders attempt to physically intervene to stop the restraint of George Floyd. For the majority of the time, the bystanders maintained a discrete distance from the officers, with the boundary demarcated by the edge of the sidewalk. That boundary was initially established in a directive from officer Lane to Charles McMillan at 20:19:08 (Lane’s body cam) to “get off the (road).” At that time McMillan was the only bystander present—none of the other significant bystanders had yet arrived on scene—and he did not immediately comply with the directive. The bystanders could first be understood to have formed a “crowd” at approximately 20:21:41 (CCTV footage) when Funari and Frazier, who are both recording on their phones, move past McMillan to stand at or just back from the edge of the sidewalk. Officer Thao turns to his left to orient to the now positioned group of four bystanders. He gestures briefly with his right hand, but no utterance is audible, before he turns back to orient to his fellow officers and George Floyd.

The first transgression of the boundary between bystander and police spaces occurred at 20:25:15 when Williams stepped off the sidewalk and onto the road. By this time Williams had been on scene for 2 min and 17 s and had made 38 intervention utterances, the majority of which had been declarative statements and assessments directed to the officers regarding the inappropriateness and illegitimacy of the form of restraint to which Floyd was being subject. As Williams stepped onto the road, officer Thao raised his left arm, pointed to Williams, and moved toward Williams. First Funari and then Williams stepped back, with Williams having stepped back onto the sidewalk by 20:25:19. As Williams stepped back, officer Thao’s arm dropped; this occurred in overlap with the utterance “get back all (.) jus- just get back on the sidewalk.” Simultaneously, and captured on Frazier’s phone footage, officer Chauvin reached to his belt and drew his can of pepper spray. This was noticed by Frazier who declared “he got mace (.) he got mace.” Funari, Frazier, and her cousin all took several steps back from the edge of the sidewalk, but Williams remained at its edge. Through these verbal and nonverbal actions, the edge of the sidewalk was explicitly established as the boundary between the bystanders and the officers, and any transgression of that boundary was clearly treatable as problematic and warranting a response from officer Thao. Over the next 3 min and 40 s, there were seven further such incursions. To these officer Thao responds on five occasions with verbal and/or nonverbal actions to reinforce the now-established boundary between bystander and officer spaces; on the other two occasions, the incursions are momentary and are quickly self-corrected by the bystander in question.

There is a degree of collective action in the bystanders’ movement. On four occasions multiple bystanders move from the sidewalk onto the road, and all such occasions are responded to by officer Thao with directives to return to the sidewalk. None of these movements are verbally coordinated among the bystanders. Rather one bystander transgresses the boundary and others follow suit. What motivates the transgression is not always clear, on at least two occasions the movement seems to be to get a better line of sight, and angle for the video recording of the officers’ treatment of George Floyd. On others the transgression seems intended to precipitate conflict with officer Thao. Throughout, the bystanders’ gestures are relatively minimal, there are points and gestures toward George Floyd and the officers, and these often accompany the assessments and imperatives, that is, they orient the recipient to the circumstances being assessed or in which they are being directed to intervene. Movements onto the road by the like of Williams and Martin are associated with very minimal, passive body language, and their hands are clasped in front of them or are by their sides; any resistance that they are presenting is nonverbally signaled to be of the passive kind.

### Critical Moments

Despite this clear and sustained evidence of verbal and physical bystander intervention, the bystanders were ultimately unsuccessful in saving the life of George Floyd. However, it does not follow that this was the inevitable outcome. As we have already established, the general disposition of the officers—in line with the deontic order—was to ignore or refuse to engage with bystanders (beyond crowd control). However, it was always possible that a particular kind of bystander intervention, or an intervention at a particular juncture, could have been taken up by the officers. If there were moments in the interaction where police officers were seen to take up some of the concerns of the bystanders—then this would be evidence that the bystanders had managed to break through the deontic barrier.

With that in mind, we explored the transcript for any evidence that there might have been some uptake of bystander concerns by any of the four officers. We identified five occasions on which this happens—all attributed to one officer, officer Lane. By looking closely at the timing of the sequence of utterances, we see five occasions wherein Lane makes a verbal intervention (toward his fellow officers), which aligns with a recent bystander intervention. Four out of five of these alignments happen after bystander assessments about the welfare of George Floyd or the legitimacy of police actions. Lane either suggests a change to the physical position of George Floyd or offers an assessment of Floyd’s well-being that aligns with the bystanders’ concerns. The fifth occasion followed an imperative from Genevieve Hansen to the officers to “check for a pulse”; Lane aligned with that imperative by asking his fellow officers “you got one?” Sadly, these breakthrough alignments are either ignored completely by fellow officers, rejected explicitly by Chauvin, or are not picked up supportively by the other officer in earshot, officer Keung. On each occasion, officer Lane does not press his inquiry a second time. Unlike the pattern of the bystander utterances, there is no repetition. These moments of alignment between bystander interventions and officer concerns are fleeting and easily extinguished, but they provide evidence that the actions of the bystanders *do* have an effect on how the officers are required to evaluate their policing.

### Transparency and Openness

Regarding ethics this research complies with the American Psychological Association and the British Psychological Society Ethical Principles for Psychologists and Code of Conduct. The study received ethical approval from Lancaster University’s Faculty of Science and Technology Ethics Committee (Ref: FST-2023-3657). With regard to preregistration, because our analysis was dependent on what could be achieved by data integration and the subsequent transcription, our analysis was exploratory and not confirmatory. Therefore, no aspects of the study were preregistered. Regarding materials, a full breakdown of video sources and a temporal timeline of video capture are publicly available on the Open Science Framework at https://osf.io/gdn9t/. No artificial intelligence-assisted technologies were used in this research or the creation of this article.

## Discussion

The most striking thing to note from the analysis is how much bystander intervention occurred and how willing the bystanders were to persevere in circumstances, which were both distressing and frustrating. This is very much in keeping with the body of evidence from research using CCTV data, which shows that levels of intervention in these kinds of violent, public space events are high (Levine et al., 2011; Liebst et al., 2019; Philpot et al., 2020). It is also important to remember that the bystanders were strangers to each other, and to George Floyd, and had no preexisting bonds of affiliation to draw on. The fact that bystander intervention emerged spontaneously among strangers, led to cooperation and coordination, and revealed a range of intervention strategies provides a sharp contrast to the usual trope of the reluctant or indifferent bystander (Lurigio, 2015).

It is also apparent that, in emergencies of this kind (where potential challenges to police violence are inoculated by the deontic order), bystanders structured their verbal interventions around the use of assessments. By opening with assessments (rather than interrogatives or imperatives), bystanders sought to navigate the power differential between themselves and the officers. Assessments (and declaratives) were used in an attempt to create legitimate grounds for behavior change among the police officers. The case for intervention is built on an appeal to epistemics rather than demands or requests. The bystanders come together by publicly articulating what they “know” (that George Floyd is in distress and that the police behavior is illegitimate). They then try repeatedly to get the officers to engage with this version of events. This analysis of the ways in which the bystanders respond to the constraints of the deontic order contributes to an emerging literature on how power relations are negotiated in talk. This includes deontic authority in the workplace (Wåhlin-Jacobsen & Abildgaard, 2020), planning meetings (Stevanovic & Peräkylä, 2012), therapeutic encounters (Nanouri et al., 2022; Ong et al., 2021), and bystander intervention itself (Ran & Huang, 2019).

Despite the ultimate failure of the bystanders to save George Floyd’s life, there is some evidence that their interventions did have an impact. On five occasions, officer Lane picks up bystander concerns and represents them to fellow officers. It is significant that Lane responds to assessments and declaratives (rather than interrogatives, imperatives, or insults) suggesting that these are of a form more suited to uptake by the more powerful group, perhaps because they do not directly or explicitly challenge the deontic order. However, it is also significant that, where the bystanders are persistent in representing their interventions (they repeat strategies despite being ignored—before switching to an alternative strategy), the interventions raised by Lane are quickly extinguished. Despite their ultimate failure, there are clear indications of “critical moments” wherein the deadly trajectory could have been changed. In these moments, the officers were asked to reflect on the concerns of the bystanders by one of their own. The moments are precipitated by the success of the bystanders in breaking through the deontic order. The failure to save George Floyd’s life results from the inability of the officers to revisit the duty of care that these moments provided.

It is notable that our analysis uncovers very little by way of “pleading” in the bystander verbal interventions. Bystanders do not “beg” the police or frame their interventions by way of pleas for “mercy.” While it is always harder to account for an absence rather than a presence in the data, it seems plausible to argue that this absence is also tied to the bystander’s understanding of the workings of the deontic order. Appealing to the better nature of the officers—from a position as a supplicant—seems to be a less favorable strategy than attempting to get the police to redefine the nature of the event unfolding in front of them.

### Limitations of Our Analysis

While the article offers important new insights into the way bystanders intervene in police violence, we recognize that this is a case where the ultimate outcome is a death at the hands of the police. Had we been able to work with an example wherein bystander interventions could be more clearly associated with a survival of police violence, then we might have been able to make a more straightforward case for the success of particular intervention strategies. However, we could not identify an analogous case wherein the “optimal capture” (i.e., the public release of the CCTV, body-worn camera footage, and the multiple bystander films) was available. It is the very notoriety provided by the lethal outcome in the George Floyd case that makes our analysis possible in the first place. However, even if we were able to find such a case, we would be wary of attempting some kind of comparison. The strength of the case study method is to allow for a detailed examination of a particular process rather than a comparison of outcomes. Robust claims of that nature cannot be made on an *N* of two. In the case of the murder of George Floyd, we clearly show that intervention strategies can have an *effect*. There is evidence (in this particular case) that bystanders were able to get at least one officer (Lane) to acknowledge their concerns. Lane then communicates this to other officers. The timings of Lane’s actions can be linked to the interventions of bystanders. We argue that this is clear evidence that bystander strategies can be effective in breaching the deontic order that can make officers impervious to bystander interventions. In other words, our analysis shows that the bystander “message” is heard—even if that does not result in behavioral outcomes that change the course of events. The fact that the officers did not use this opportunity to reevaluate and change course does not undermine the case that bystanders were able to engineer interventions that had an effect. What we still need to explore in more detail (and with different data) is how to understand the barriers and facilitators of a constructive police response when engaging with bystanders. This is an important but slightly different question.

At the same time, by engaging in detail with the bystanders in the event itself, we recognize that there is a broader bystander context, which is not addressed in the article. There is the history of police violence, deaths of Black Americans, and subsequent protests (including the killings of Eric Garner, Michael Brown, Tamir Rice, Walter Scott, Alton Sterling, Philando Castile, Stephon Clark, and Breonna Taylor in the 6 years before George Floyd’s murder). There are also the “bystanders” who witnessed this event vicariously through videos on social media. Moreover, there are the protests in America and across the world that brought millions of people out onto the street in the name of the “Back Lives Matter” movement. The behavior described in this article will clearly be shaped by that history and will also have contributed in the aftermath to broader cultural discourses about police–community relations in general and bystander behavior in particular. However, our microlevel focus on the nature of the immediate verbal and physical *interactions* precludes any contribution to these more macrolevel questions.

### Implications for Research and Training

Overall, this analysis has clear implications for research on bystander intervention more generally and for the way bystanders and police officers can be trained to improve the safety of individuals caught up in police activity. The analysis demonstrates, for the first time, some of the discursive intervention strategies that are available to bystanders in these particular kinds of emergency events. This focus on the way speech acts are understood in relation to the power relations in emergency events takes us beyond the usual “4D”s (direct, distract, delegate, and delay) advocated by bystander training programs like “Green Dot” (see Coker et al., 2015). A focus on the discursive and conversational levels of analysis is increasingly being translated into practical intervention training (see, e.g., Stokoe, 2020).

In terms of practical implications for improving the success of bystander interventions, there are lessons for both bystander and police officer training. Promoting successful bystander intervention involves much more than getting people to speak up (Levine et al., 2020). That is just the start and not the most challenging problem. To facilitate successful bystander intervention, we need to know more about what intervention techniques might work and how they will play out in the intergroup context.

In other words, successful bystander intervention is an outcome of both positive intra-group processes (among the bystanders themselves and among the police officers themselves) and positive intergroup communication (between bystanders and officers).

From a police training perspective, it is clear that we need to train officers in a way that goes beyond a focus on procedure (cf. Tyler, 2006). Officers need to be trained to engage in conversational interaction with bystanders around welfare and a duty of care and not to treat bystanders as a potential threat to be managed. Moreover, when it comes to training officers to challenge unprofessional behavior by colleagues, existing “active bystandership” training (like the Active Bystandership for Law Enforcement Project; see Kurtz & Tucker, 2023) or the Ethical Policing is Courageous (New Orleans Police Department; see Aronie & Lopez, 2017) need to be expanded to recognize the role that bystanders can play in shaping the trajectory of events.

One powerful legacy of the sustained, creative, and resilient efforts of the bystanders to the George Floyd murder is that they require us to rethink our traditional approaches to how we understand bystander behavior.

## Figures and Tables

**Table 1 tbl1:** Bystander Utterance Type, Their Definitions, Examples, and the Frequencies With Which They Were Addressed to Either GF or One or More of the PO

Utterance type	Example	Frequency count
Declarative statements (utterances that provide information or state facts)	“the man gonna win” (McMillan) “he is human” (Williams)	GF oriented = 5 PO oriented = 17
Assessments (utterances that convey some evaluation of either George Floyd’s well-being or his treatment by the officers)	“now you’re worse” (McMillan) “he’s not responsive right now bro” (Williams)	GF oriented = 17 PO oriented = 48
Interrogatives (utterances that are phrased as questions, both real and rhetorical)	“does he have a pulse?” (Hansen) “d- what do you think that’s okay?” (Williams)	GF oriented = 3 PO oriented = 40
Imperatives (utterances that tell the addressee to do something)	“check his pulse right now and tell me what it is” (Hansen)	GF oriented = 3 PO oriented = 44
Insults (utterances that disrespect or demean the addressee)	“you’re a fucking pussy ass dude bro” (Williams)	GF oriented = 0 PO oriented = 9
*Note*. GF = George Floyd; PO = police officers.		

**Figure:**
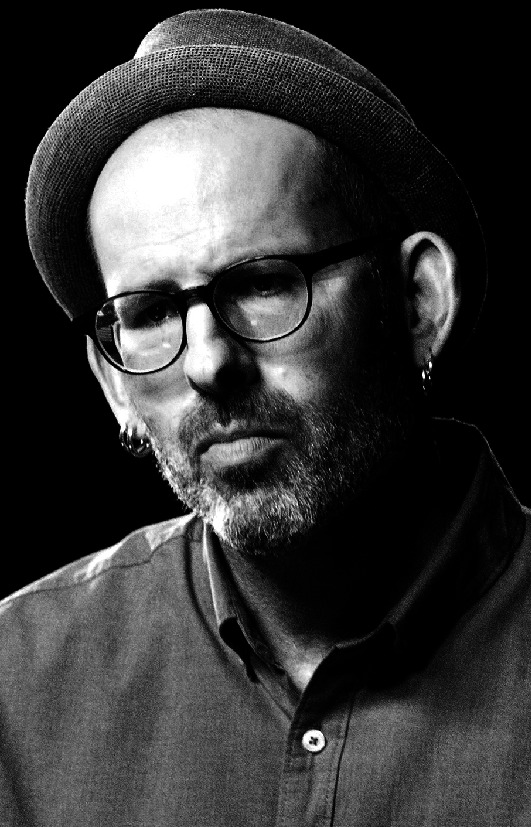
Mark Levine

**Figure:**
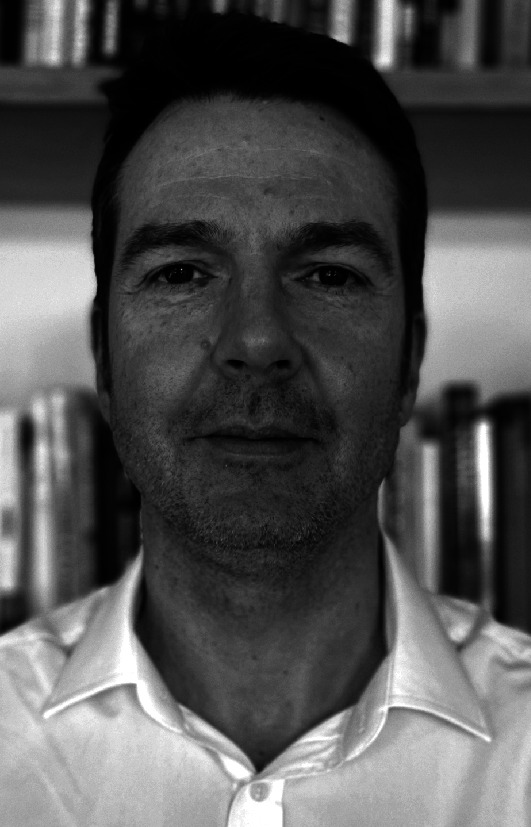
Chris Walton

**Figure:**
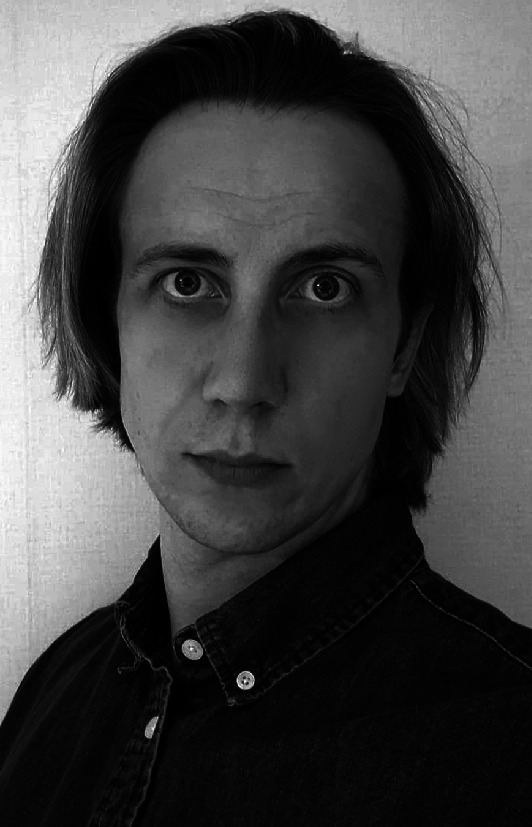
Richard Philpot

**Figure:**
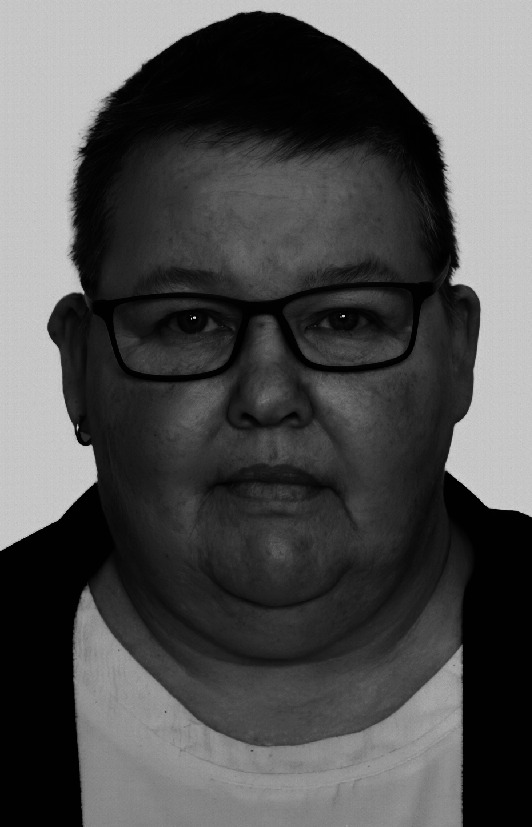
Tina Keil
